# Impact of Anthropogenic Activities on the Dissemination of ARGs in the Environment—A Review

**DOI:** 10.3390/ijerph191912853

**Published:** 2022-10-07

**Authors:** Małgorzata Czatzkowska, Izabela Wolak, Monika Harnisz, Ewa Korzeniewska

**Affiliations:** Department of Water Protection Engineering and Environmental Microbiology, Faculty of Geoengineering, University of Warmia and Mazury in Olsztyn, Prawocheńskiego 1, 10-720 Olsztyn, Poland

**Keywords:** antibiotics, antimicrobial resistance, anthropogenic pressure, wastewater, sewage sludge, landfills, biogas plants, animal husbandry, agriculture

## Abstract

Over the past few decades, due to the excessive consumption of drugs in human and veterinary medicine, the antimicrobial resistance (AR) of microorganisms has risen considerably across the world, and this trend is predicted to intensify. Many worrying research results indicate the occurrence of pools of AR, both directly related to human activity and environmental factors. The increase of AR in the natural environment is mainly associated with the anthropogenic activity. The dissemination of AR is significantly stimulated by the operation of municipal facilities, such as wastewater treatment plants (WWTPs) or landfills, as well as biogas plants, agriculture and farming practices, including animal production and land application of manure. These activities entail a risk to public health by spreading bacteria resistant to antimicrobial products (ARB) and antibiotic resistance genes (ARGs). Furthermore, subinhibitory concentrations of antimicrobial substances additionally predispose microbial consortia and resistomes to changes in particular environments that are permeated by these micropollutants. The current state of knowledge on the fate of ARGs, their dissemination and the complexity of the AR phenomenon in relation to anthropogenic activity is inadequate. This review summarizes the state-of-the-art knowledge on AR in the environment, in particular focusing on AR spread in an anthropogenically altered environment and related environmental consequences.

## 1. Introduction

**Figure 1:**
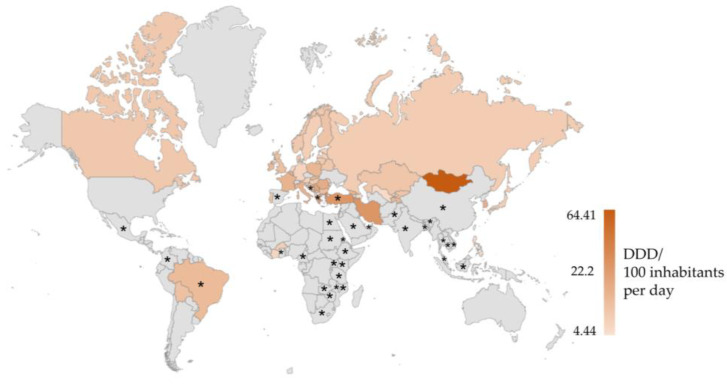
Map of total antibiotic consumption in 2015–2016, based on data acquired by the World Health Organization (WHO) from 65 countries [4]. DDD—daily defined dose. The black asterisk (*) marks countries where the consumption of antibiotics is not properly controlled, and antimicrobials can be purchased without a prescription [6,7,8,9,10].

Antibiotics have been used for decades in the pharmacotherapy of bacterial and fungal infections. The discovery of antibiotics is counted among the most important achievements in the history of medicine [1]. Pharmaceuticals are broadly used in human and veterinary medicine. However, their frequent and unwarranted consumption raises a serious concern, compounded by the lack of social awareness of the consequences of excessive administration of drugs [2]. Klein et al. [3] informed that the global consumption of antibiotics increased by 65% in the years 2000–2015, and the predicted rise in drug consumption by 2030 peaks at 200% relative to the year 2015. Based on the data collected by the World Health Organization (WHO) in 65 countries around the world, the average highest antibiotic consumption was noted in the Eastern Mediterranean Region, while the lowest one was in the African Region and the Region of the Americans [4]. The daily defined dose (DDD) in Mongolia, over the whole period analyzed, soared to 64.41 per day (Figure 1). Antibiotics can be excreted by both humans and animals in an unchanged form or as products of their metabolism, regardless of the dose taken; therefore intensive drug consumption in the world increases the pool of released drugs in the environment [5].

Unused antibiotics, which are supposed to be returned for safe disposal, are often mixed with other waste and reach landfills so that their presence is detected in landfill leachate (LL) [11,12]. The management of LL generated in landfills often consists of the purification of LL together with municipal wastewater at wastewater treatment plants (WWTPs) [13]. Moreover, the wastewater delivered to WWTPs is also found to contain antimicrobial substances, used in the treatment of people and animals, and most often discharged to the environment in an unchanged form [14]. After the wastewater treatment process is completed, wastewater is delivered to water receivers, such as rivers and other surface water bodies, that are directly tied to the natural environment. The presence of antibiotics in wastewater is a serious problem and an environmental threat in the circular wastewater economy [15]. In turn, the high supply of antibiotics in veterinary medicine contributes to the presence of antimicrobial substances in the waste generated by intensive animal breeding, for example, in slurry. Slurry, with the antibiotics and antibiotic metabolites it contains, most often undergoes stabilization, after which it can be discharged into soil environment [16], and then, with run-offs from farmland, it can pollute water bodies. A popular slurry stabilization method is anaerobic digestion (AD) with the generation of biogas. The digestate obtained by AD is also used as fertilizer, and the micropollutants it may contain permeate into the soil environment, groundwater and surface waters [1], thereby increasing the pool of pollutants in the soil and water environments, as well as enabling the circulation of antibiotics in these ecosystems.

Depending on the class of pharmaceuticals, it is estimated that from 40 to 80% of a dose of a drug taken by people or animals are excreted with stool and urine in an unchanged, that is active form [17,18]. The most popular antibiotics used globally belong to the classes of quinolones, macrolides, beta-lactams, and aminoglycosides [19]. Pharmaceuticals from the classes of tetracyclines and sulfonamides are the antibiotics broadly used in veterinary medicine, whereas macrolides, penicillin and fluoroquinolones are most often administered in human medicine [20]. Drugs not metabolized in human or animal organisms enter the environment and threaten soil and water ecosystems and plants. The presence of antibiotics in different ecosystems, even in small concentrations, can cause a series of consequences adverse to the environment. These medications can influence the biodiversity of microorganisms and, most importantly, the pool of antimicrobial resistance genes (ARGs) found in microbiomes settled in a given ecosystem, which can be associated with a direct or indirect risk to public health [21]. Antimicrobial resistance (AR) relates to elevated hospitalization and mortality rates, and new zoonotic diseases caused by antibiotic-resistant bacteria (ARB). This is a serious problem concerning the health of people and the welfare of animals [22]. Health care authorities from the United States of America [23] and the European Union [24] estimate that at least 35,000 and 33,000 premature deaths per year due to infections caused by ARB occur in the USA and Europe, respectively. Uncontrolled exposure of many environments to antibiotics leads to the persistent selective pressure on microorganisms inhabiting these ecosystems. Moreover, ARB and ARGs can be spread with air, water and through links of trophic chains [25,26,27].

**Figure 2:**
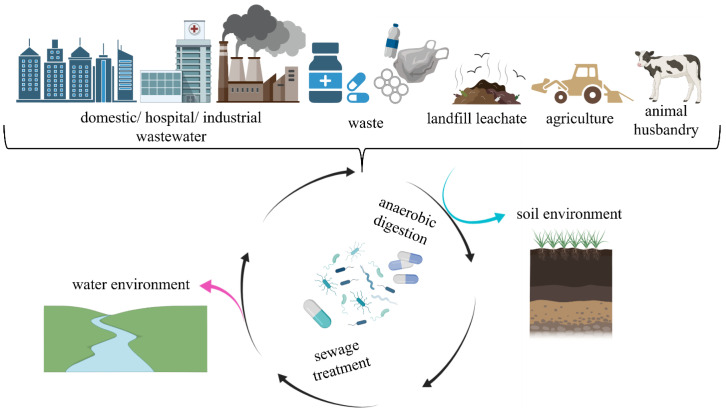
The potential origin and fate of ARGs in the environment.

Drug resistance is a well-described global phenomenon with grievous environmental and epidemiological consequences [28]. Human activity largely contributes to the anthropologically shaped environment becoming a reservoir of ARB and ARGs [29]. The dissemination of AR in the environment is mainly due to the operation of municipal facilities, such as WWTPs [30,31] and landfills [13,32], as well as the sector of agriculture [33,34], animal rearing [35,36] and biogas plants [37]. These areas have a considerable accumulation of ARB, which can transfer ARGs between each other (Figure 2).

Antibiotics, ARB and ARGs raise a growing concern among researchers and institutions dealing with protecting public health and the environment, and there has been a global discussion on AR for years. The extent of environmental pollution caused by the excessive consumption of drugs, both in human and veterinary medicine, is enormous and therefore gives rise to serious worries [38,39,40,41]. In 2006, based on the information about AR determinants, the concept of resistome was developed, understood as a set of all ARGs among pathogenic and commensal microorganisms inhabiting a given ecological niche [42]. It was also concluded that anthropogenic activities directly shape or indirectly affect the environmental AR, while ARGs can be transmitted between people, animals and the environment. In 2016, during the General Assembly of the United Nations, heads of the UN member states admitted that it was necessary to reinforce systems to monitor infections caused by ARB and amounts of antimicrobial medications used in medicine, veterinary medicine and plant production [43].

Numerous research papers dedicated to the spread of AR have laid the foundation for further studies under the program called ‘One Health’, to gain insight into this issue in the context of human medicine, veterinary medicine and the broadly understood environment in a holistic approach. Reduction of the dissemination of AR is one of the principal assumptions of the ‘One Health’ strategy. Another objective is to promote the monitoring of the environment and conduct joint research by scholars from many fields of science, such as public health, veterinary medicine and environmental protection. This strategy also highlights the strong dependence between the health state of animals and people and the condition of the environment they occupy together.

Importantly, in 2019, the European Commission launched the European Green Deal [44], which promotes measures ‘to increase the efficient use of resources in order to achieve a clean and circular economy, to restore biodiversity, and to reduce pollution.’ In the coming years, it is therefore expected that the consumption of chemical fertilizers will decrease while the use of organic fertilizers, based on livestock and poultry manure, will increase [45]. Meanwhile, monitoring the microbiological contamination of natural fertilizers by ARB and ARGs is insufficient. It is expected that the ongoing international programs devoted to the reduction of AR will enable the implementation of information and research programs and the legal regulations serve to strengthen the control measures and prevent the spread of AR.

The main objective of this study has been to review the current state of knowledge on the impact of anthropogenic activities on the presence of antibiotics and the spread of ARB and ARGs in the environment. The paper describes key reservoirs of antibiotics, ARB and ARGs in the environment and the hotspots involved in their release due to human activity. In addition, the current knowledge on the effects of antibiotic residues on the environment has been reviewed, and the broad consequences of environmental pollution with pharmaceuticals and ARGs have been described to gain a better insight into these issues and support future research.

## 2. Materials and Methods

This study has developed a protocol to specify the research questions, criteria for inclusion/exclusion, data sources and scientific literature search engines. The authors adhered to the checklist of the Preferred Reporting Items for Systematic Review and Meta-Analyses Extension for Scoping Reviews (PRISMA-ScR) to carry out the review.

### 2.1. Data Sources

In compliance with the PRISMA guidelines, the articles were selected according to the four criteria: (i) identification, (ii) screening studies, (iii) eligibility, and (iv) inclusion. The SCOPUS, PubMed, and Google Scholar scientific literature databases were surveyed to find reviewed papers published from 1 January 2010 to 27 September 2022.

### 2.2. Search Strategy

The strategy employed in the search is illustrated Supplementary Materials Figure S1. The keywords used in the search strategy were: (“Antibiotic Resistance” OR “antibiotic resistance genes” OR “anthropogenic”) AND (“antibiotic resistance genes” OR “antibiotic resistance” OR “co-selection” OR “heavy metals” OR “microplastic”). These were tailored to each database.

**Figure 3:**
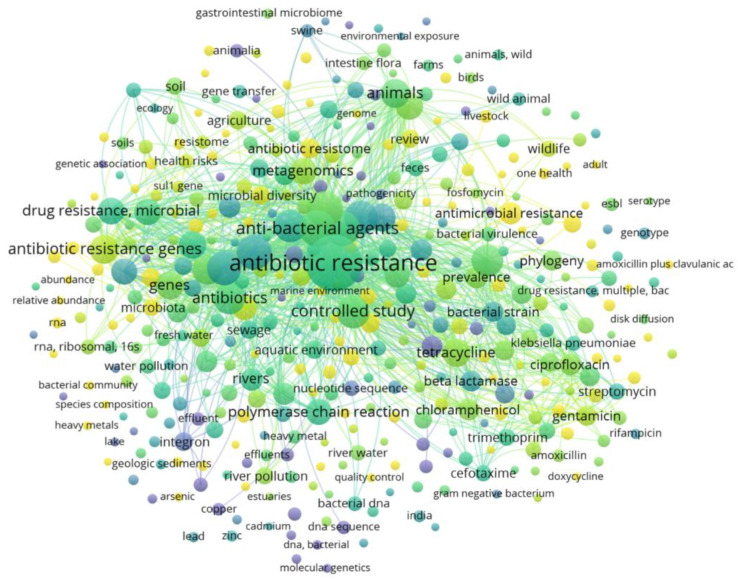
A keyword co-occurrence map, considering papers containing “antibiotic resistance” as a keyword. Circle size is proportional to the number of co-occurrences of a particular keyword and clustering by color is based on patterns of co-occurrence among multiple keywords in the published articles. The map was created with VOSviewer (v1.6.16; 2020, Centre for Science and Technology Studies, Leiden University, The Netherlands).

A preliminary search was conducted of the published scientific literature related to the subject of this study to identify the keywords to be employed in the advanced search. The keywords for the search are presented in Figure 3. Complementary searches (including forward and backward citation searches of included articles) were conducted to further locate eligible articles that were not identified in the databases search. In addition, a reference list of articles was checked manually so as to find adequate scientific publications for this review of literature data. After filtering the literature, 225 scientific publications were selected for this review article. Figure S2 shows the publications used for this review, grouped by publication year.

The references identified through the searched terms were imported into Mendeley (Copyright © 2021 Mendeley Ltd., Amsterdam, The Netherlands), and duplicates were removed. The articles were analyzed by reviewing the titles and abstracts in line with our inclusion and exclusion criteria, and the articles selected for this review were read in full text.

## 3. Results

### 3.1. Municipal Facilities as Reservoirs of ARGs

The growing global human population and the continual development of local communities are associated with the need to manage huge amounts of wastewater and solid waste. The municipal amenities responsible for this task, such as WWTPs, waste sorting facilities and disposal sites, are an important source of ARB, ARGs and residues of antimicrobial substances, which can further permeate the environment. Based on the review of the literature data, we identified three main reservoirs of ARGs associated with the municipal economy: WWTPs, landfills and biogas plants.

#### 3.1.1. WWTPs

High usage of water by medical care institutions leads to the generation of large quantities of hospital wastewater and sewage. In developed countries, hospitals generate from 400 to 1200 L of wastewater per patient, while in developing countries, this amount ranges between 200 and 400 L [46]. Wastewater from the health care sector is characterized by the presence of a wide array of microorganisms of special clinical importance, including ARB-carrying ARGs. In addition, hospital wastewater also contains antimicrobial substances used in the treatment of patients [47,48]. In view of the ever-growing consumption of drugs and the development of the health care system, the generation of large amounts of hospital wastewater and its proper management are an enormous challenge in environmental engineering [46].

According to the literature data, although hospital wastewater is treated in hospital wastewater treatment plants (HWWTPs), it is still a reservoir of antibiotics, ARB and ARGs. The subsequent delivery of hospital wastewater to municipal WWTPs is an additional source of promoting the exchange of genetic structures between microorganisms, i.e., horizontal gene transfer (HGT). The process of HGT plays a major role in the dissemination of AR among bacteria [49] and can be realized by three well-studied mechanisms; (1) transduction (transfer of genetic material between bacteria via bacteriophages) [50], (2) transformation (changing the bacterial genotype through extracellular DNA acquire) [51] or (3) conjugation (exchange of conjugative plasmids between physically attached bacteria). Conjugation is commonly observed in nature, even among distantly related microorganisms [19]. 

The bacteria present in hospital wastewater are particularly predisposed to HGT processes [52]. Yao et al. [53] analyzed the occurrence of antibiotics, ARB and ARGs in wastewater from three hospitals, each using different wastewater treatment processes, including disinfection. These authors noted an incomplete effectiveness in the removal of antibiotics through the processes carried out in HWWTPs, as well as a relatively high abundance of ARGs in treated wastewater, which is then conveyed to WWTPs. Moreover, concentrations of some ARGs encoding the resistance to beta-lactams increased after the treatment in HWWTPs (*bla*_OXA-1_, *bla*_OXA-10_ and *bla*_TEM-1_). The wastewater treated in HWWTPs was also distinguished by the high counts of pathogenic or opportunistic bacteria of the genera *Acinetobacter*, *Klebsiella*, *Aeromonas* and *Pseudomonas*. These results confirmed the co-occurrence of antibiotics, ARB and ARGs in treated hospital wastewater. When such wastewater is delivered to WWTPs, it enriches the pool of ARB and ARGs in incoming wastewater. As reported in literature references, ARB and ARGs in wastewater from hospitals can be two to nine orders of magnitude higher than in typical municipal wastewater [54].

The main goal of the processes carried out in WWTPs is to lower organic matter content in wastewater and reduce the counts of microorganisms, including pathogenic ones. The structure of the microbiota in influent wastewater can vary and the wastewater treatment processes induce changes in the number and biodiversity of microorganisms, which may contain ARGs. Differences in the structure of the microbiotas characteristic for the wastewater delivered to WWTPs and for treated wastewater are presented in Table 1.

Antibiotics are mentioned among the micropollutants present in wastewater entering WWTPs (Table 2). It has been confirmed that there are effective, chemical and physicochemical methods for potentially eliminating antibiotics and other AR determinants from wastewater but because of the operating costs, these methods are not widely used [59,60]. As the technological processes most often employed in WWTPs do not unfortunately include technologies specifically designed to remove pharmaceuticals from wastewater, these pollutants eventually end up in surface water bodies together with treated wastewater. Effluent wastewater from WWTPs can be, therefore, one of the major sources of ARB and ARGs in water ecosystems [61,62,63,64].

Wastewater treatment plants are among the human-made facilities that create conditions particularly suitable for the occurrence of processes of exchange of genetic structures, including ARGs, between microorganisms dwelling in wastewater being treated in these facilities. At the same time, WWTPs promote the increased selection of bacteria possessing specific ARGs [73]. High counts of microorganisms and subinhibitory concentrations of antimicrobials present in influent wastewater contribute to the transfer of ARGs between microorganisms due to the so-called selection pressure and, consequently, to their spreading in wastewater on an enormous scale [74]. Dissemination of ARGs among microorganisms is also associated with mobile genetic elements (MGEs), such as plasmids, conjugation transpons and integrons. Mobile genetic elements allow the capture and expression of exogenous genes [75]. MGEs also facilitate the transfer of ARGs between microorganisms. Moreover, this transfer can also be stimulated by the presence in the ecosystem of such antibiotics as beta-lactams or tetracyclines, popular in human and veterinary medicine [76]. Of particular concern is the fact that even when selective pressure is absent or weak, MGEs can be transferred between microorganisms [77].

Many researchers have analyzed ARB and ARGs in wastewater sampled at WWTPs [74,78,79,80,81,82,83,84,85,86,87]. It has been confirmed that the general population’s seasonal intensity of drug consumption affects both the concentrations of ARGs in wastewater and the extent of their further transmission to the environment [78]. Wastewater has been observed to contain clinically significant strains of bacteria characterized by drug resistance [79,80]. It has also been found that despite the high percent reduction of ARB and ARGs resulting from wastewater treatment, considerable amounts of these micropollutants are still discharged into the environment together with treated wastewater [78,79]. Furthermore, it has been determined that ARB and ARGs can be transferred in bioaerosol from wastewater to the mucus membrane of the upper respiratory tract among the WWTPs employees, thereby increasing their exposure to infectious agents [80]. The types of ARGs whose presence has been detected in influent and effluent wastewater sampled at WWTPs and HWWTPs are summarized in Table 3.

Wastewater treatment plants can receive thousands of m^3^ daily of wastewater, which can carry an immense load of various kinds of pollutants. The processes carried out in WWTPs aiming to remove impurities from wastewater typically comprise the pre-treatment stage, where most of the suspended solids should be removed. The next stage is the biological treatment of wastewater, where primarily the activated sludge technology is worth noting. Wastewater is then conveyed from biological treatment chambers to a secondary sedimentation tank, where it is separated from activated sludge. Some of the sludge is recirculated back to the bioreactors, while the remaining amounts are removed [90]. The initial sludge generated in the early stage of wastewater treatment and the excess sludge from bioreactors compose a pool of sewage sludge that requires proper disposal and management.

Impurities present in influent wastewater reaching WWTPs, including microbiological pollutants and residues of antimicrobial substances, accumulate in sewage sludge. There are many reports [73,83] confirming that as a result of the presence of antibiotics as well as ARB and ARGs in effluent wastewater from WWTPs, their occurrence is also observed in sewage sludge. The types and concentrations of antibiotics and ARGs, in addition to the abundance and composition of microbial assemblages present in sewage sludge, can vary and is directly dependent on the quality of wastewater received by WWTPs, and on the type of processes involved in the wastewater treatment. Data concerning the occurrence of particular micropollutants in sewage sludge are collated in Table 4.

Considering the ongoing dissemination of AR in the environment, it is significant to acknowledge the fact that quantities of sewage sludge produced by WWTPs are growing constantly, reaching hundreds of millions of Mg annually across the whole world. However, not all countries keep statistics on the production of wastewater and sewage sludge. For example, in India, the second country in the world in terms of population (18.04%) and the seventh in size, the data concerning this subject are very limited or fragmentary. It is known that the production of wastewater in India in 2014–2015 was 62,000 million m^3^ a day, while the wastewater treatment capacity in the same time period was slightly over 23,000 million m^3^ a day [104]. There are no available data on the production and handling of sewage sludge in that country. 

Another Asian country, China, is one of the leading producers of sewage sludge, generating an amount of 11 × 10^6^ Mg annually [105]. Less than 30% of this amount is used as fertilizer, 26.7% is incinerated, and 20% is deposited on landfills [106]. In 2019, around 317 thousand Mg of dry matter of sewage sludge was produced in Australia; 70% of this mass fertilizes agricultural land, and 26% is used for soil reclamation purposes. The remaining 6% is deposited in landfills or discharged into the ocean [107].

In the USA, the annual output of sewage sludge in 2019 reached 4.7 × 10^6^ Mg, of which more than half ended up in landfills [108]. On the other hand, data pertaining to sewage sludge production in South America are scanty. In Brazil, the largest and most populous country on this continent, many municipalities do not possess adequate technologies for wastewater treatment, as a result of which untreated wastewater is discharged to surface water bodies, posing a direct threat to the environment [109]. In 2015, Brazil was inhabited by over 204.5 million people, but only 98 million had access to sewers [110].

In the European Union, the largest sewage sludge is produced by Germany, Spain, Italy and France. Table 5 shows data on sewage sludge production in the EU member states, according to the information provided on the Eurostat website [111]. However, these data are fragmentary because they do not illustrate the whole scale of sewage sludge generation each year by all the EU member states. In the last set of data for the year 2019, some of the largest sewage sludge producers, such as Spain and Italy, are missing [111]. Nearly half of the sewage sludge produced in the EU is used in agriculture and enters soils. Slightly less than 13% are applied for soil reclamation, and almost 9% are deposited in landfills [112].

Because of the content of organic substances and nutrients, the agricultural use of sewage sludge is a preferred option in many countries [112,113]. Using sewage sludge as a fertilizer is a solution to the problem of its utilization but considering the risk of spreading AR that it involves, this practice creates a real threat to the environment and public health.

Due to the legal restriction on depositing sewage sludge binding in many countries, to manage this type of waste, it is first submitted to stabilization and then used in agriculture as a valuable source of nitrogen and phosphorus, for making compost and for reclamation of degraded land [114]. Disposal of sewage sludge is most often achieved by composting or AD. However, the application of aerobically or anaerobically treated sewage sludge for soil fertilization may trigger serious ecological problems, especially in the context of polluting soil with antibiotics [45]. Antibiotic residues in sewage sludge can appear in a wide range from ng to 100 mg kg^−1^ of the dry matter of sewage sludge [39]. Analysis of the efficiency of sewage sludge stabilization does not include checking the presence of drugs or the counts of ARB and ARGs. Thus, the pollutants contained in sewage sludge eventually enter the soil environment, which creates a risk of an adverse impact on the physical, chemical and biological properties of soils [15]. Among the consequences of soil fertilization with stabilized sewage sludge containing residues of pharmaceuticals, there are changes in the structure of the soil’s microbiome and resistome, and possible transmission of ARGs between microorganisms inhabiting a given ecosystem.

#### 3.1.2. Landfills

Depositing waste on landfills is a widespread, global practice to dispose of and stabilize the solid fraction of municipal waste, known as MSW (Municipal Solid Waste) [115]. The total amount of MSW gathered in landfills reaches hundreds of millions of Mg annually. The method of landfilling MSW is economically competitive relative to other waste management methods, which is why it is the most common solution used in developing countries [116]. Nevertheless, landfilling makes an important contribution to waste management even in highly developed countries. For instance, 20, 104, 19, 55 and 330,000 Mg of waste daily were deposited in Australia, Denmark, Spain, Sweden and China, respectively, in 2017 [117]. Although, in some countries, the number of active municipal landfills is on the decrease, there are still thousands of active landfills which are planned to be closed in a decade or a few decades. The major problem in waste management, however, is not the quantities of landfilled MSW but the inadequate handling thereof. Particularly in developing countries, nearly 90% of the solid fraction of municipal waste is landfilled without any pretreatment [116].

Landfills contain a wide array of pollutants, including heavy metals or complex organic and inorganic compounds [116]. Moreover, the lack of social awareness concerning risks due to environmental pollution with antibiotics contributes to the wrong handling of unused or expired medicines, which may directly stimulate the increasing content of these contaminants in landfills. Landfills are generally considered to be the site for storing both medicines and illegal clinical waste, used nappies and pet excreta. Antibiotics, like any pharmaceuticals, should be stored properly, and when expired or not used completely, they should be disposed of correctly. However, insufficient social awareness often leads to the incorrect handling of antibiotics, which means that many unused antibiotics end up in landfills. The presence of antibiotics can exert pressure on communities of bacteria, affecting the occurrence of ARB and ARGs [118]. The widespread, excessive and improper use of antibiotics observed nowadays raises serious concerns about the prevalence of ARGs, which are frequently detected in landfills and in LL.

The main problem with landfills is that antibiotics, ARB and ARGs can be transferred to the environment via LL [119]. Water seepage through waste deposited in landfills leads to the leaching of various types of pollutants. The following can be distinguished: suspended substances, dissolved substances and substances originating from the decomposition of waste, as well as microorganisms, including pathogens. One of the biggest challenges connected with the operation of waste disposal plants is to handle LL generated in landfills in an environmentally sound manner [120]. A popular solution is the treatment of LL together with municipal wastewater at WWTPs [121], which results in the additional enrichment of municipal waste with antibiotics, ARB and ARGs [61]. Table 6 presents the specification of antibiotics detected in LL.

Many research papers have analyzed the presence of antibiotics, ARB and ARGs in LL. It has been noted that the occurrence of some classes of antibiotics in LL can correlate with the abundance of a local population [116]. It has been observed that concentrations of particular antibiotics in LL from landfills disused for years can remain very high, in excess of the Predicted No Effect Concentrations (PNEC) relative to the AR selection [123]. It has also been demonstrated that LL can be a substantial reservoir of ARGs and MGEs [13,116,124]. The presence of MGEs can be closely correlated with the abundance of ARGs, and the frequency of ARGs and MGEs can correlate additionally with the concentration of particular elements, including heavy metals [116]. Moreover, while some authors have observed significant differences in the distribution of ARGs in LL samples from different landfills [116], others have not noticed any evident regional pattern of distribution of these micropollutants [124]. Wang et al. [13] noted that the process of LL treatment is effective in the removal of ARGs. The research results provided by these scholars confirmed the effect of LL on the water resistome in a river to which treated LL was discharged. This suggests the risk of spreading AR determinants in the environment due to the discharge of treated LL to surface water bodies.

The literature data show that both landfills and LL play a role in the significant pools of antimicrobial substances (Table 6), and ARGs (Table 7) in the environment, thus predisposing AR to uncontrolled development in the environment.

#### 3.1.3. Biogas Plants

Due to the human population growth, progressing urbanization and intensification of agriculture, the amounts of organic waste generated worldwide have turned into a huge burden on the natural environment. In order to produce alternative, eco-friendly energy and to reduce quantities of landfilled waste, many biogas plants, both agricultural ones and operating at WWTPs and landfills, have been launched in recent years. Nowadays, there are about 50 million micro-bioreactors and a total of 132,000 small, medium and large bioreactors operating worldwide. This number is continually increasing, and the potential for the further development of the biogas plant sector is immense and found in every country [128,129]. Anaerobic digestion (AD) has become an attractive technology for the stabilization of organic residues, in which waste is ‘a renewable resource’ as it can be reused for generating new products and biofuels [130]. Methane fermentation creates great potential for the production of an environmentally friendly fuel such as biogas [131]. Biogas produced by AD can be converted into a more efficient biofuel, such as biomethane [132]. The data collected by the European Biogas Association show that the number of biogas plants producing biomethane in Europe increased in two years from 483 (in 2018) to 729 (in 2020). At present, biomethane is produced in 18 European countries, and the largest producers are Germany (232 biogas plants), France (131) and the United Kingdom (80) [133].

Different types of organic waste can be submitted to AD, for example, animal feces [128], by-products from the food processing industry [134] and the animal feed industry [135], sludge from WWTPs [136], or post-harvest residues [137], which are degraded and converted into biogas and the process’s by-product called digestate [138]. Because of its high content of valuable nutrients, digestate can be used as a fertilizer in plant production [139]. However, to ensure sanitary, environmental and food safety, prior to using digestate for agricultural purposes, it must achieve proper quality in terms of both the concentrations of nutrients and the content of pollutants, e.g., heavy metals and pathogens. Digestate obtained by AD is especially hazardous in this regard, as it may contain antibiotics, ARB and ARGs, thus contributing to the dissemination of AR in the environment [96,140,141] (Table 8).

There are many studies attesting to the fact that the intensive use of antibiotics makes antimicrobial substances enter organic substrates converted by AD, as a result of which such substances then appear in digestate [96,103,143,148,149]. The presence of drugs from the classes of tetracyclines, sulfonamides, and fluoroquinolones has been detected in digestate from poultry litter [125], sewage sludge [96], and bovine slurry [147]. What is more, antibiotics present in AD processed substrates, especially ciprofloxacin, norfloxacin [150], and tetracycline, can demonstrate high resistance [146]. The occurrence of drugs in digestate can exert selective pressure on microorganisms, which creates a potential pathway for acquiring and spreading ARGs, which have been detected in digestate in many studies [45,147]. The scientific literature also confirms that bacteria from the phyla *Firmicutes*, *Bacteroidetes*, and *Proteobacteria* are the major ARGs carriers, hence when there is no selective pressure, the succession of the mentioned microorganisms may affect the transfer and dissemination of ARGs between microorganisms [96,151,152].

The AD process conducted in line with the current technological possibilities does not guarantee complete removal of antibiotics, ARB or ARGs. Furthermore, the risk of ARGs emission to the environment is also affected by the storage time of digestate before it is used in agriculture. It has been found that a 30-day storage time of digestate decreased the total relative number of ARGs, while resulting in an increase in the counts of particular ARGs sub-types, including *tet*M, *tet*X, *tet*Q, *tet*S, *erm*F, and *sul*2 [149].

The release of antibiotics, ARB, and ARGs present in digestate to the environment threatens the public health and distorts the microbiological balance in soils and waters. The challenge technology engineers are facing is to develop new, more efficient technologies and strategies for intensive management in order to enhance the removal of ARGs at all stages of AD.

### 3.2. The Impact of Agriculture and Animal Husbandry on the Presence of Drugs, ARB and ARGs in the Environment

Processes of obtaining plant and animal products by plant breeding and growing and by animal breeding and rearing are closely related to circular economy. The animal excreta from animal production are the main type of waste generated in agriculture. The biggest challenge concerning the handling of this waste arises from its content of veterinary antimicrobial drugs. Antibiotics found in farm animal feces determine the patterns of resistance to pharmaceuticals among the microorganisms present in manure. Because the most popular way of managing such waste as manure is to use it for fertilization, antibiotics, ARB, and ARGs can accumulate in soil and in crops. The exposure of the soil environment to manure containing antimicrobial substances leads to a selective advantage of ARB in the environment. Moreover, antibiotic therapy used in animal production can have a significant influence on the occurrence of antimicrobial substances, ARB and ARGs in food offered to consumers.

#### 3.2.1. Agriculture

As mentioned in Section 3.1.3, drugs used in agriculture enter the environment mainly with manure, commonly used all over the world for fertilization of soils. Degradation of antibiotics in an animal organism depends on the type of an antimicrobial substance. As much as over 80% of the dose of an antibiotic administered to an animal can be excreted with urine and stool in an unchanged form or as metabolites [18]. It has been demonstrated that antibiotics show strong inclination towards adsorption to manure, and the degree of adsorption depends on the state of matter [153]. Ezzariai et al. [154] noted that residues of antibiotics in animal feces reached amounts as high as 136 mg kg^−1^ of dry matter. Other researchers recorded high concentrations of sulfamethazine and tetracycline in manure-based fertilizer samples, which were 5650 and 1920 mg kg^−1^, respectively [155].

Antibiotics, ARB, and ARGs can also be spread in the environment with wastewater from animal farms and with runoffs from agricultural lands, which is a source of hazard. Areas in the vicinity of rivers and other surface water bodies are often used as agricultural land, where vegetables, cereals and other crops are grown. They are also used by livestock for grazing or drinking water. Moreover, irrigating arable fields with treated wastewater is a common practice, although it adds to the dissemination of micropollutants in agricultural habitats [156]. In liquid matrices, drugs often appear in amounts below the level of detectability, which means that the actual amounts of antibiotics in the soil and water environment may be underestimated. Even despite their low concentrations, antibiotics continue to be bioavailable and their influence on the environment and on AR can be substantial. Agricultural activity contributes to the spread of antibiotics, the acquisition of ARGs by microorganisms, and the dissemination of ARB and ARGs on a broad scale, also through the food chain. The presence of ARGs has been recorded in plantations of crops grown for human consumption [18,157,158]. Consumption of many unprocessed, raw leafy and non-leafy vegetables, root vegetables, sprouts or fruits could be the cause of human exposure to microorganisms, including ARB, inhabiting such types of food [21,159]. Scientific research completed in recent years has shown the presence of antibiotics, ARB and ARGs in agricultural products, for example seeds [160], parsley roots [158], or lettuce [156,161] (Table 9).

Endophytic bacteria colonizing plant tissues may possess ARGs and be present in different plant organs: roots, stems, leaves or fruits. The most diverse microbiome of plants is found in the roots, which is a consequence of their immediate contact with soil [164,165]. It has been demonstrated that the exposure of crops to antibiotics may promote their growth with the simultaneous accumulation of antimicrobials in plant tissues [160]. The degree to which plants absorb antibiotics depends on various biotic and abiotic factors and on the type of crop. Cereals and fruits are less prone to absorbing pollutants than leafy and root vegetables [166]. Moreover, the presence of antibiotics induces an increased frequency of ARB among the total endophytic bacteria [160]. Additionally, fresh plant products can contain opportunistic microorganisms, including ones of the genera *Klebsiella* and *Enterobacter*, whose presence has been detected on such vegetables as cabbage, pepper or tomatoes [159,167,168]. Furthermore, some ARB determined in soil and manure show phylogenetic similarity to human pathogens (e.g., of the genus *Acinetobacter*), thereby raising the probability of genetic exchange between microorganisms [169]. In order to constrain the effect of soil fertilization on increasing the diversity of the resistome of both the soil and crops, there is no doubt that specific legal regulations must enter into force, especially in developing countries.

#### 3.2.2. Animal Husbandry

In order to meet the demand for animal-origin food, antibiotics have become an indispensable element of livestock rearing. Models of using antibiotics in animal production differ depending on the world’s region, country’s policy and type of production. In industrial countries, meat consumption has been slightly decreasing in recent years while growing rapidly in developing countries, where access to veterinary antibiotics is not regulated, and the knowledge on AR is insufficient [20]. Some antimicrobial drugs are forbidden in developed countries but can still be used in most developing states [170]. Although some countries have limited the use of antibiotics in livestock exclusively to medical purposes (including some EU member states, in compliance with 1831/2003/EC of 2016), these pharmaceuticals continue to be used in excess in many regions around the world, where intensive animal production is carried out (the USA, China, Russia, Indie and Republic of South Africa). It is estimated that antibiotic therapy in livestock production in the USA corresponds to around 80% of the total consumption of antimicrobial substances in this country. Moreover, most of the antimicrobials used for this purpose are also administered in human medicine [171,172].

At present, approximately 30 different antimicrobial classes are used in livestock production across the world. Among the drugs administered to farm animals, there are mainly macrolides, beta-lactams and tetracyclines [173]. In European countries, the total quantity of applied antibiotics converted per kg of animal body is no less than 20 up to 188 mg. Most antibiotics are used in the breeding and rearing of swine and poultry, and the average dose in the world is 172 and 148 mg, respectively, per kg of animal body weight. In turn, the same dose for cattle is around 45 mg per kg of body weight [171]. In 2013, the use of antimicrobials in animal production administered in order to treat diseases and as growth stimulants reached 420 mg annually in the United Kingdom and 14,600 mg annually in the United States. For comparison, in the same year, 2013, the consumption of antibiotics in livestock production in China peaked at 84,500 mg [174]. Unfortunately, the administration of drugs to animals is inevitably associated with the risk of their presence in food products of animal origin.

Thermal treatment of products of animal origin can reduce the risk of consuming antibiotics these products may contain, such as sulfonamides, fluoroquinolones and tetracyclines, but cannot eliminate drugs from the class of beta-lactams. The persistence of the latter class of antibiotics can lead to a situation where residues of these antibiotics are found in thermally treated milk and in dairy products made from such milk, being a threat to the health of consumers [20]. Antimicrobial substances have been detected in milk [20], sheep meat [175], poultry meat [176] and beef [177] (Table 10).

The lack of adequate veterinary supervision and the administration of subtherapeutic doses of antibiotics are the major factors contributing to the spread of AR in livestock populations. Contemporary animal husbandry is often characterized by high livestock density and routine administration of antibiotics, which may predispose it to the emergence of new zoonotic pathogens resistant to antibiotics. The presence of ARB in animals reared for meat and in food products made from such animals has been documented all over the world. Methicillin-resistant *Staphylococcus aureus* (MRSA) and strains of *Escherichia coli* resistant to colistin, as well as *E. coli* resistant to carbapenems, have been detected in swine. Species of *Campylobacter jejuni* and *Campylobacter coli* resistant to ampicillin, streptomycin and tetracycline have been isolated from chickens, while multidrug-resistant strains of *Pseudomonas aeruginosa* and *Acinetobacter baumannii* have been isolated from swine, poultry and cattle [182,183,184,185,186].

Antibiotic-resistant bacteria can be transferred from farm animals to humans directly via food, such as meat, fish, eggs and dairy products [187]. Numerous outbreaks of food infections caused by ARB, including strains of *Staphylococcus aureus* and *Escherichia coli* and various species of the genera *Enterococcus*, *Aeromonas* and *Salmonella*, are linked to food made from farm animals have been reported worldwide [188,189]. Moreover, the transmission of resistant strains can occur via different routes, also between animals of different species, through direct contact with other animals, or their saliva, feces or blood, which contain ARB [170]. This creates a risk of transmitting ARB and ARGs from animals to humans and of human pathogens acquiring resistance to the classes of antibiotics used in both veterinary and human medicine.

A way to decelerate the spread of AR in animal production is to reduce or optimize the use of antibiotics in animal husbandry. Additionally, it is recommended to improve the hygiene and animal housing conditions as well as the quality of feeds, which will have a direct influence on the welfare and health of animal herds and flocks, thus eliminating superfluous antibiotic treatments. Moreover, other prophylactic measures are recommended, such as inoculations and supplementation of feeds with pro- and prebiotics as well as bioactive compounds (e.g., antimicrobial peptides) [190].

### 3.3. Co-Selection of ARGs by Other Anthropogenic Pollutants

#### 3.3.1. Heavy Metals

Heavy metals (HMs), the most common of which are lead (Pb), zinc (Zn), mercury (Hg), nickel (Ni), cadmium (Cd), copper (Cu), chromium (Cr) and arsenic (As), are widely distributed in the environment. These pollutants are detected in wastewater [191], sewage sludge [92,192], LL [193], manure [18,194] and in fertilized soil [195]. Heavy metals are not biodegradable, can be toxic and carcinogenic, and pose a serious threat to lifeforms and the environment. Some of the HMs detected, even in scant amounts, may be dangerous [191,196].

The half-lives of HMs are estimated to be hundreds or even thousands of years. One of the longest half-lives characterizes copper, which is widely used in various industrial sectors [197]. The presence of HMs in wastewater shows an increasing tendency with the development of human and industrial activities. These pollutants accumulate in sewage sludge, and wastewater loaded with the presence of HMs continues to the aquatic environment, threatening the ecosystem and human health [191,198]. The occurrence of these micropollutants in the waste stream at the landfill, as well as in LL, creates a risk that they enter soil and surface waters [199]. The presence of HMs is also found in provender for cattle, pigs, and poultry [200]. Moreover, some studies showed that pig feces had higher concentrations of zinc (941.1 mg kg^−1^) and copper (137.6 mg kg^−1^) compared to their provender (139.8 and 31.5 mg kg^−1^, respectively). The use of fertilizers based on sewage sludge and manure containing HMs leads to their accumulation in the soil and creates an additional, long-term selection pressure on microorganisms [201].

The presence of HMs and antibiotics in water and soil environments, as a result of various anthropogenic activities, leads to the exposure of microorganisms to both kinds of pollutants [197]. The connection between HMs and AR proliferation has been analyzed in wastewater and solid waste [202,203,204], agriculture [194,205,206] and industrially contaminated environments [207,208]. Significant incidence of ARB and ARGs from HMs and antimicrobial co-contaminated environments suggests that exposure of microorganisms to HMs pollution co-selects AR [209]. The co-selection of AR occurs when microorganisms harbor two different resistance genes towards antimicrobials and HMs (co-resistance) or one gene which is responsible for tolerance to antibiotics and HMs (cross-resistance) [209,210,211]. A variety of HMs at concentrations found in different environments have the ability to co-select ARB and resistance plasmids. If resistance genes for both types of compounds are located on the same plasmid, exposure to HMs can also promote the HGT of AR [210]. Horizontal gene transfer impacts microbial evolution and leads to the dissimilation of ARGs among both environmental and clinical microorganisms [212]. Long-term exposure of microorganisms to HMs can lead to changes in biodiversity and abundance of ARGs, and the co-selection of ARGs caused by the presence of these micropollutants is perceived as another threat to the environment [208,210,213].

#### 3.3.2. Microplastics

The continuously growing production and use of plastics have resulted in an increase in the stream of this fraction of waste all over the world. Pollution of the environment from microplastics (MP), which are plastic debris smaller than 5 mm in diameter, is a common and global problem that will aggravate in the future [212]. Concerns about the presence of MP in various environments are compounded by their ability to adsorb many chemicals, including antibiotics and HMs. Moreover, microplastic debris provides a hydrophobic surface to support the formation of biofilms by microorganisms. For this reason, MP is an anthropogenic vector for the large-scale transport of ARB and ARGs [209,212,214,215]. Particles of MP, together with ARB and ARGs, have been identified in both wastewater [216] and LL [217], as well as in the air [218], soil [219] and river and sea water [220].

Municipal facilities, such as WWTPs and landfills, are considered hotspots for antibiotics, ARB, and ARGs, as well as MP [216]. Municipal wastewater contains MP from clothing and personal care products [221,222]. It is estimated that 80–90% of MP in wastewater is retained in the sludge, but the remainder enters the aquatic environment along with the treated wastewater and permeates the soil with soil-applied stabilized sewage sludge [223]. Microplastics present in the waste fraction in a landfill and in LL may further infiltrate into the soil and water environment [216]. Microplastics migrating into the environment, carrying antibiotics, ARB, and ARGs, affect changes in microbial communities and resistomes. Interestingly, the type of polymer plays a role in the transfer of AR determinants; some studies have shown that polyethylene (PE) has greater transport potential compared to polypropylene (PP) [217]. Moreover, the presence of additional micropollutants such as antibiotics or HMs may increase the pool of ARGs carried on MP [216]. It is worrying that both antibiotics and HMs, as well as MP, can intensify the development of AR among microorganisms and stimulate HGT [224,225]. The particles of MP constitute a vector for many micropollutants, additionally supporting the co-selection of ARGs based on the presence of HMs [209].

## 4. Conclusions

In sum, it should be concluded that the anthropogenic environment and all objects it contains have a direct influence on the presence of antibiotics and the spread of ARB and ARGs in the environment. The facilities associated with municipal infrastructures, such as WWTPs and landfills, but also biogas plants and agriculture, including plant and animal production, are key reservoirs of antibiotics, ARB and ARGs. The anthropogenic activity enables a huge pool of antimicrobials to enter the environment, which leads to their uncontrolled consumption by people and animals, also due to the improper disposal of unused drugs. An incomplete metabolism of antibiotics and the fact that antibiotics permeate various environments contribute to the selective pressure, thereby facilitating an increase of the ARGs pool among microorganisms. Many scientific reports indicate that ARGs are omnipresent in a variety of anthropogenic environments, and their range of occurrence is very wide. A significant role in the dissemination of ARGs is played by MGEs and HGT processes, which take place in various environments, especially the ones at WWTPs. Both antibiotics and ARB and ARGs are released to the water environment together with discharged treated wastewater and with LL, as well as via surface runoffs from arable fields and farm buildings. Furthermore, the soil environment is most often enriched with these micropollutants as a consequence of the fertilization of fields with stabilized waste from municipal facilities and the agricultural sector. 

The information collated in this review proves that further research is needed to answer the question of how to prevent the proliferation of ARGs in the environment in order to reduce the risk of AR acquisition by microorganisms. Moreover, the important role of other anthropogenic pollutants such as HMs and MP, which can additionally co-select AR and intensify its development among microorganisms, was highlighted. A better insight into the role of anthropogenically transformed environments in the dissemination of AR is necessary for undertaking specific legislative initiatives and effectively reducing the mentioned phenomenon.

## Figures and Tables

**Table 1:** The dominant bacterial phyla in WWTPs influent and effluent.

Type of Wastewater	Country of Research	Dominant Bacterial Phyla (Percentage)	Reference
influent	Germany	*Firmicutes* (52.2%), *Proteobacteria* (37.8%), *Bacteroidetes* (4.9%), *Actinobacteria* (2.2%)	[55]
effluent		*Proteobacteria* (54.8%), *Bacteroidetes* (15.7%), *Firmicutes* (14.3%), *Planctomycetes* (2.9%), *Actinobacteria* (2.6%), *Verrucomicrobia* (2.1%)	
influent	China	*Firmicutes* (54%), *Proteobacteria* (34%), *Actinobacteria* (7%), *Bacteroidetes* (2%)	[56]
effluent		*Proteobacteria* (44%), *Actinobacteria* (13%), *Bacteroidetes* (12%), *Firmicutes* (6%)	
influent	China	*Proteobacteria* (51.3%), *Firmicutes* (16.4%), *Actinobacteria* (4.7%), *Verrucomicrobia* (1.8%)	[57]
effluent		*Proteobacteria* (31.2%), *Firmicutes* (1.1%), *Actinobacteria* (8.1%), *Verrucomicrobia* (2.5%)	
influent	Poland	*Proteobacteria* (55.13%), *Firmicutes* (25.6%), *Bacteroidetes* (6.3%), *Actinobacteria* (10.65%)	[58]
effluent		*Proteobacteria* (39.06%), *Firmicutes* (10.1%), *Bacteroidetes* (19.62%), *Actinobacteria* (26.26%), *Verrucomicrobia* (2.54%)	

**Table 2:** Antimicrobial substances most frequently detected in the influent wastewater collected from the WWTPs.

Antimicrobial Class	Antimicrobial Substance	Concentration [ng L^−1^]	Reference
beta-lactam	amoxicillin	232–5698	[65]
	ampicillin	306–4120	[65]
	penicillin G	120–2230	[66]
fluoroquinolone	ciprofloxacin	475–913	[67,68]
	ofloxacin	130–730	[66,69],
imidazole	metronidazole	4.83–161.0	[67,70]
macrolide	clarithromycin	904–7.3 × 10^6^	[67,68,71]
	erythromycin	5–2300	[71,72]
sulfonamide	sulfamethoxazole	387–5.3 × 10^6^	[67,68,71]
	sulfadiazine	326–1072	[71]
tetracycline	tetracycline	26.23–4160	[66,70]
	doxycycline	16.44–97.91	[70]

**Table 3:** Types of ARGs detected in the wastewater collected from WWTPs.

Type of Wastewater	Type of Samples	ARGs	The Relative Abundance of ARGs (Number of Copies Normalized against 1 mL of Sample or Gene *16S r*RNA)	Ref.
Hospital wastewater	Influent	*bla*_GES-1_	from 4.6 × 10^−5^ to 1.4 × 10^−3^	[53]
		*bla*_TEM-1_	from 8.6 × 10^−5^ to 1.3 × 10^−4^	
		*bla*_OXA-1_	from 7.5 × 10^−4^ to 1.2 × 10^−3^	
		*qnr*S	from 8.2 × 10^−7^ to 1.7 × 10^−5^	
		*qn*A	from 5.5 × 10^−6^ to 1.2 × 10^−6^	
	Effluent	*bla*_GES-1_	from 6.3 × 10^−5^ to 1.6 × 10^−3^	
		*bla*_TEM-1_	from 6.4 × 10^−6^ to 5.4 × 10^−4^	
		*bla*_OXA-1_	from 5.1 × 10^−4^ to 1.0 × 10^−4^	
		*qnr*S	from 5.3 × 10^−7^ to 1.3 × 10^−6^	
		*qnr*A	from 9.3 × 10^−7^ to 2.6 × 10^−6^	
			gene copies/*16S r*RNA	
	Effluent	*bla*_TEM_, *erm*B, *qnr*S, *sul*1, *tet*W	range from 10^4^ to 10^7^ gene copies in 1 mL of sample	[81]
	Effluent	*bla*_NDM_, *bla*_KPC_, *bla*_CTX-M_, *bla*_SHV_, *sul*1, *aac(6′)-Ib*	range from 10^4^ to 10^9^ gene copies in 1 mL of sample	[88]
	Effluent	*bla*_SHV_, *bla*_TEM_, *bla*_CTX_, *bla*_OXA_, *bla*_KPC_, *bla*_NDM_, *erm*B, *sul*1, *sul*2, *tet*A, *tet*B, *tet*C, *tet*O, *tet*W, *tet*M	range from 10^−5^ to 10^−2^ gene copies/*16S r*RNA	[89]
Municipal wastewater	Influent	*bla*_TEM_, *tet*A, *sul*1	In winter: from 2.56 × 10^4^ to 1.19 × 10^9^ In autumn: from 2.23 × 10^2^ to 3.56 × 10^7^ gene copies in 1 mL of sample	[78]
	Influent	*bla*_SHV_, *tet*A, *aac(6′)-Ib-cr*	from 10^4^ to 10^8^ gene copies in 1 mL of sample	[82]
	Effluent	*tet*M,	1.9 × 10^4^	[83]
		*tetO*	7.7 × 10^4^	
		*tetW*	1.0 × 10^4^	
		*sul*1	5.4 × 10^6^	
		*sul*2	7 × 10^5^	
			gene copies in 1 mL of sample	
	Influent/effluent	*aad*A, *str*B, *bla*_OXA_, *erm*F, *sul*2 *tet*W, *qac*H	na ^a^	[84]
	Influent/effluent	*amp*R, *bla*_CIT_, *bla*_CTX-M_, *bla*_FOX_, *bla*_GES_, *bla*_IMP_, *bla*_NPS_, *bla*_OXA_, *bla*_SHV_, *bla*_TEM_, *bla*_VIM_, *mec*A, *erm*B, *erm*F, *mac*B, *mef*A, *mph*, *mel*, *gyr*A, *par*C, *qnr*, *dfr*, *sul*1, *sul*2, *sul*3, *tet*, *acr*B, *acr*D, *mdt*, *mex*	na	[74]
	Influent/effluent	*bla*_TEM_, *qnr*A, *qnr*S, *sul1, erm*B, *intI*1	na	[85]
	Effluent	*qnr*S, *bla*_TEM,_ *sul*1, *erm*B, *bla*_OXA-58,_ *tet*M, *int*l1	na	[86]
	Effluent	*qnr*D, *qnr*S, *erm*A, *erm*B, *tet*A, *tet*Q, *sul*1, *sul*2	na	[87]
	Influent	*bla_TEM_, erm*B, *qnr*S, *sul*1, *tet*W	range from 10^4^ to 10^7^ gene copies in 1 mL of sample	[81]
	Effluent	*bla_TEM_, erm*B, *qnr*S, *sul*1, *tet*W	range from 10^2^ to 10^4^ gene copies in 1 mL of sample	

^a^ na—data not available.

**Table 4:** Antimicrobials, microorganisms and ARGs detected in sewage sludge.

Antimicrobial Class and Antimicrobial Substances	Ref.	Dominant Bacterial Phyla and Genera	Ref.	ARGs	Ref.
**Fluoroquinolone:** ofloxacin [0.5–7950 μg kg^−1^], norfloxacin [75.5–21,335 μg kg^−1^], ciprofloxacin [<1–4720 μg kg^−1^], enrofloxacin [<1–77.5 μg kg^−1^], sarafloxacin [<1–14.6 μg kg^−1^], fleroxacin [≤1840 μg kg^−1^], lomefloxacin [≤502 μg kg^−1^] **Sulfonamide:** sulfadiazine [≤51.9 μg kg^−1^], sulfamethoxazole [≤17 μg kg^−1^], sulfapyridine [≤47.7 μg kg^−1^], sulfamethazine [≤11.7 μg kg^−1^], sulfamerazine [≤3.7 μg kg^−1^] **Macrolide:** erythromycin [≤55.8 μg kg^−1^], roxithromycin [≤342 μg kg^−1^], tylosin [≤33.8 μg kg^−1^], spiramycin [≤13.3 μg kg^−1^] **Tetracycline:** oxytetracycline [174.2–36,650 μg kg^−1^], tetracycline [101–2943 μg kg^−1^], chlortetracycline [5.95–3843.7 μg kg^−1^], doxycycline [127.45–2104.2 μg kg^−1^]	[91,92,93,94,95]	*Proteobacteria* (*Acinetobacter*, *Aeromonas*, *Alcaligenes*, *Comamonas*, *Brevundimonas*, *Methylobacterium*, *Stenotrophomonas*), *Bacteroidetes* (*Bacteroides*, *Cloacibacterium*, *Paludibacter*, *Sphingobacterium*, *Flavobacterium*), *Firmicutes* (*Clostridium*, *Bacillus*) *Actinobacteria* (*Propionibacterium*, *Mycobacterium*), *Acidobacteria*, *Saccharibacteria*, *Spirochaetes* (*Treponema*)	[96,97,98,99,100]	*aad*A, *bla*_TEM_, *bla*_OXA_, *tet*C, *tet*G, *erm*B, *erm*C, *erm*F, *sul*1, *sul*2, *bex*A, *qep*A, *aac(6′)-Ib-cr, tet*M, *bla*_CTX-M_, *bla*_IMP_, *qnr*S, *aac(3)-1*, *dfr*A1, *dfr*A5, *dfr*A7, *dfr*A12,	[96,100,101,102,103]

**Table 5:** Sewage sludge production and disposal in selected countries in 2019. Based on data from Eurostat, 2022.

Country	Production of Sewage Sludge (Thousand Mg)
Germany	1749.86
Poland	574.64
Austria	233.56
Romania	230.59
Hungary	227.89
Czech Republic	221.09
Norway	141.35
Albania	96.20
Ireland	58.63
Slovakia	54.83
Lithuania	39.94
Slovenia	34.80
Estonia	24.94
Latvia	24.18
Croatia	20.65
Malta	9.69
Serbia	9.60
Bosnia and Herzegovina	9.50
Luxembourg	8.89 (e) ^a^

^a^—estimated.

**Table 6:** Summary of antimicrobial substances and their concentration in landfill leachates, based on data collected by Yu et al. [122].

Antimicrobial Class	Antimicrobial Substance	Concentration [ng L^−1^]
macrolide	azithromycin	from 13.5 to 50.2
	erythromycin	from 12.0 to 39,800.5
	roxithromycin	from 7.8 to 4745.8
beta-lactam	cefotaxime	from 3.1 to 72.3
	cephalosporin	from 11.77 to 537
	penicillin G	from 22 to 160
fluoroquinolone	ciprofloxacin	from 4.9 to 4482.5
	norfloxacin	from 25.9 to 21,033.33
	ofloxacin	from 8.7 to 190,000
sulfonamide	sulfadiazine	from 15.3 to 29,208
	sulfamethoxazole	from 0.7 to 8488
	sulfamonomethoxine	from 9.8 to 2750
tetracycline	doxycycline	<228
	oxytetracycline	<3245.0
	tetracycline	from 0.2 to 19,000

**Table 7:** Types of ARGs detected in landfill leachate.

Dominant Microorganisms	ARGs	The Relative Abundance of ARGs (Number of Copies Normalized against *16S r*RNA or ngDNA)	Ref.
Genera: *Acholeplasma*, *Aminivibrio*, *Candidatus Cloacamonas*, *Petrimonas*, *Sedimentibacter*, *Tissierella*	*sul*1, *sul*2, *erm*F, *aad*A, *bac*A,	>1.0 × 10^−1^/*16S r*RNA	[124]
na ^a^	*qnr*A	1.1/*16S r*RNA	[116]
	*qnr*B	1.13 × 10^−5^/*16S r*RNA	
	*qnr*D	4.95 × 10^−6^/*16S r*RNA	
	*bla*_OXA10_	3.86 × 10^−4^/*16S r*RNA	
	*pen*A	10^−6^–10^−5^/*16S r*RNA	
na	*tet*O	from 4.1 × 10^−5^ to 4.2 × 10^−2^/*16S r*RNA	[125]
	*tet*W	from 5.7 × 10^−5^ to 4.9 × 10^−3^/*16S r*RNA	
	*bla*_TEM_	from 3.7 × 10^−5^ to 3.9 × 10^−2^/*16S r*RNA	
	*sul*1	from 4.5 × 10^−5^ to 3.1 × 10^−2^/*16S r*RNA	
	*sul*2	from 1.4 × 10^−4^ to 6.2 × 10^−2^/*16S r*RNA	
Phyla: *Proteobacteria*, *Firmicutes*, *Chloroflexi*, *Actinobacteria*, *Bacteroidetes*, *Acidobacteria*	*sul*1	5.6 ± 0.9 log10/ng DNA	[126]
	*aad*A1	5.5 ± 0.8 log10/ng DNA	
	*bla*_CTX-M_	4.1 ± 0.7 log10/ng DNA	
na	*tet*M	from 2.99 × 10^−3^ to 2.16 × 10^−2^/*16S r*RNA	[127]
	*tet*X		
	sul1		
	sul2		

^a^ na—data not available.

**Table 8:** Data on the presence of antibiotics, ARB and ARGs in various digestate materials from AD process.

**Source**	**Country**	**ARB ^a^ in Samples**	**AAs of ARGs ^b^ (in 1 g_D_^c−1^)**	**Antibiotics Persistent in Sample (µg g^−1^)**	**Ref.**
sewage sludge digestate	Poland	na ^d^	*bla*_OXA_ and *bla*_TEM_ from 10^4^ to 10^7^; *tet*A, *tet*M and *tet*Q from 10^3^ to 10^7^; *sul*1 10^7^−10^8^; *erm*F, *lin*A and *mef*A from 10^4^ to 10^8^	0.26 of MET ^e^; 2.91 of SMX ^f^; 1.25 of CEF ^g^; 4.55 of DOC ^h^; 1.25 of OXY ^i^; 1.74 of CIP ^j^; 2.07 of NA ^k^	[96]
corn shredded, triticale, soya, cotton seeds, corn flour and fresh zoological waste digestate	Italy	na	*aac-(6′)-Ib-cr* up to 10^5^; *qnr*S up to 10^7^; *qep*A up to 10^6^	7.5 of CIP; 0.25 of SMX	[139]
sewage sludge digestate	Türkiye	na	na	1.49 of CLAR ^l^; 1.49 of AZYT ^m^; 5.03 of CIP; 5.35 of DOXY; from 0.22 to 3.63 of OXY; 2.57 of SMX; 0.07–2.52 of CHLOR ^n^; 0.03–1.30 of ERY ^o^; 6.63 of SMX; 4.34 of TRIM ^p^	[142]
cattle manure digestate	Poland	na	from 10^4^ to 10^5^ of *bla*_TEM_ and *bla*_OXA_; from 10^5^ to 10^7^ of *cfx*A; from 10^7^ to 10^9^ of *tet*A, from 10^8^ to 10^9^ of *tet*M; from 10^7^ to 10^9^ of *tet*Q; from 10^5^ to 10^8^ of *erm*F; from 10^5^ to 10^6^ of *lin*A, from 10^6^ to 10^7^ of *mef*A; from 10^7^ to 10^8^ of *sul*1; from 10^5^ to 10^7^ of *aac(6′)-Ib-cr*; from 10^5^ of 10^9^ of *qep*A; from 10^6^ to 10^7^ of *int*I1; from 10^6^ to 10^8^ of *int*I2	0.02 of MET; 4.35 of ENR; 0.24 of SMX; 9.62 of OXY; 1.63 of CHLOR; 5.07 of TET ^r^	[143]
food waste and slurry digestate	China	*Pedobacter, Fluviicola, Devosia, and Desulfatiglans*	from 10^1^ to 10^3^ of *erm*B, *tet*M, *tet*W and *int*I1; from 10^4^ to 10^6^ of *bla*_TEM_, *erm*B, *tet*M, *tet*W and *erm*F	na	[144]
dairy manure digestate	China	na	*tet*W, *sul*2 and *int*I2 > 10^9^ copies g _dry solid_ ^−1^; *tet*C, *tet*M, *tet*Q and *tet*X > 10^7^ copies g _dry solid_ ^−1^	na	[145]
swine slurries and their digestates	Spain	na	from 10^10^ to 10^11^ of *int*I1, *sul*1 and *tet*M; lower than 0,1% of *bla*_TEM_, *bla*_CTX-M-32_, *bla*_OXA-58_, *qnr*S and *mec*A.	na	[146]
dairy manure and effluent digestate	USA	na	from 10^3^ to10^4^ *sul*1, *sul*2, *tet*M and *tet*G	na	[147]

^a^—antibiotic resistance bacteria, ^b^—antibiotic resistance genes, ^c^—amount of ARGs per 1 g of digestate, ^d^—data not available ^e^—metronidazole, ^f^—sulfamethoxazole, ^g^—cefuroxime, ^h^—doxycycline, ^i^—oxytetracycline, ^j^—ciprofloxacin, ^k^—nalidixic acid, ^l^—clarithromycin, ^m^—azithromycin, ^n^—chlortetracycline, ^o^—erythromycin, ^p^—trimethoprim, ^r^—tetracycline.

**Table 9:** Antimicrobials, ARB and ARGs detected in selected food samples of plant origin.

Source	Country of Research	ARB ^a^ in Samples	AAs ^b^ of ARGs ^c^ (in 1 g_D_^d−1^)	Antibiotics Persistent in Sample	Ref.
Soil and lettuce	Australia	na ^e^	144 different ARGs to beta-lactam, aminoglycoside, macrolide-lincoside-streptogramine B (MLSB) and tetracycline from 4.37 × 10^9^ to 2.02 × 10^10^ g^−1^ (soil); from 7.45 × 10^6^ to 8.24 × 10^7^ g^−1^ (lettuce)	na	[21]
Groundwater	Poland	na	*int*I1 from 3.60 × 10^1^ to 8.73 × 10^3^; *int*I2 from 9.88 × 10^2^ to 9.73 × 10^3^; *sul*2 4.32 × 10^4^*; sul*1 1.98 × 10^4^*; bla*_TEM_ 1.58 × 10^3^*; aad*A9 1.63 × 10^1^*; dfr*A1 9.73	1.01 × 10^−2^–9.09 × 10^−2^ ng mL^−1^ SMX ^f^	[18]
Crops cultivated on manure-amended plots			*sul*2 6.54 × 10^11^; *tet*A 1.94 × 10^11^; *tet*M 2.80 × 10^10^; *sul*1 3.10 × 10^9^	na	
Parsley roots and leaves			*bla*_TEM_ parsley roots from 5.25 × 10^5^ to 1.41 × 10^7^; in parsley leaves from 3.56 × 10^5^ to 9.21 × 10^5^; *sul*1 from 1.75 × 10^6^ to 7.18 × 10^6^ (roots) and from 1.03 × 10^6^ to 3.33 × 10^6^ (leaves) *aad*A9 from 6.66 × 10^4^ to 1.06 × 10^5^ (roots).	from 2.28 ng g_dm_^−1 g^ to 6.02 ng g_dm_^−1^ of DOXY ^h^ (roots)	
Seeds of Pakchoi–vegetable endophytic systems	China	Antibiotic-resistant endophytic bacteria 10^3^ CFU ^i^·g^−1^	*tet*X, *bla*_CTX-M_, *sul*1 and *sul*2~10^−6^ copies per *16S r*RNA	na	[160]
lettuce leaves, roots, and soil,	China	na	*int*I1, *tet*W, *erm*F, *erm*X, and *sul*1 ranged from 10^2^ to 10^9^	na	[161]
soil, rhizospheric soil, broad beans, lettuce *Lactuca sativa* L. cv. Batavia, roots, leaves and beans in tomatoes *Lycopersicon esculentum* Mill.	Spain	na	*sul*1, *tet*M, *qnr*S1, *bla*_CTX-M-32_, *bla*_OXA-58_, *mec*A (except broad beans), *bla*_TEM_ ranged from 1 to 10^6^	na	[162]
carrot tuber fertilized with pig manure	China	na	*mdt*H_2, *bla*_CMY_1_, *van*SB, *sul*2, *int*I1_cli, *mex*F, *cat*B8, *flo*R, *tet*T, *aac(6′)-Ib, aadA2_3*~4.8 × 10^4^	na	[163]

^a^—antibiotic resistance bacteria, ^b^—absolute abundances, ^c^—antibiotic resistance genes, ^d^—amount of ARGs per 1 g of digestate, ^e^—data not available, ^f^—sulfamethoxazole, ^g^—dry mass, ^h^—doxycycline, ^i^—colony forming unit.

**Table 10:** The presence and concentration of various antibiotics in selected food samples of animal origin.

Source	Country of Research	ARB ^a^ in Samples	Antibiotics Persistent in Sample	References
Milk	Bangladesh	na ^b^	61.2 and 124 μg L^−1^ respectively for OXY ^c^ and AMO ^d^	[178]
Chicken, beef and pork	Republic of South Africa	na	20.7–82.1, 41.8–320.8, 65.2–952.2 and 32.8–95.6 μg kg^−1^, respectively, for SUL ^e^, TET ^f^, STREP ^g^ and CIP ^h^	[177]
Chicken and fish	Bangladesh	na	508.4 mg kg^−1^ AMO (chicken) 515.4 mg kg^−1^ AMO (fish)	[179]
Chicken	Indonesia	na	up to 275 ng g^−1^ CIP up to 242 ng g^−1^ ENRO ^i^	[176]
Broiler meat and liver	Bangladesh	*Campylobacter jejuni* i *Campylobacter coli*	10–155; 25–135 and 50–115 μg kg^−1^, respectively, for OXY, CIP and ENRO	[180]
Meat of the sea bream (*Sparus aurata*) and sea brass fish (*Dicentrarchus labrax*)	Türkiye	na	4.25 ng kg^−1^ CHLOR ^j^	[181]

^a^—antibiotic resistance bacteria, ^b^—data not available, ^c^—oxytetracycline, ^d^—amoxicillin, ^e^—sulfanilamide, ^f^—tetracycline, ^g^—streptomycin, ^h^—ciprofloxacin ^i^—enrofloxacin, ^j^—chlortetracycline.

## Data Availability

Not applicable.

## Supplementary Materials

The following supporting information can be downloaded at: www.mdpi.com/article/10.3390/ijerph191912853/s1, Figure S1: SCOPUS PRISMA flowchart showing the results of the publication’s search and screening process for this review; Figure S2: The number and date of publications used to develop this review.
