# Supplementary material for: Impact of Anthropogenic Activities on the Dissemination of ARGs in the Environment—A Review
Source: Int J Environ Res Public Health. 2022 Oct 7;19(19):12853. doi: 10.3390/ijerph191912853 (PMC9564893; doi:10.3390/ijerph191912853)
Supplement: Supplementary file 1 [file ijerph-19-12853-s001.zip › ijerph-1908225-supplementary.pdf]

Supplementary materials

Impact of anthropogenic activities on the dissemination of ARGs in the environment—A review

Małgorzata Czatzkowska, Izabela Wolak, Monika Harnisz and Ewa Korzeniewska *

Department of Water Protection Engineering and Environmental Microbiology, Faculty of Geoengineering, University of Warmia and Mazury in Olsztyn, Prawocheńskiego 1, 10-720 Olsztyn, Poland
* Correspondence: ewa.korzeniewska@uwm.edu.pl

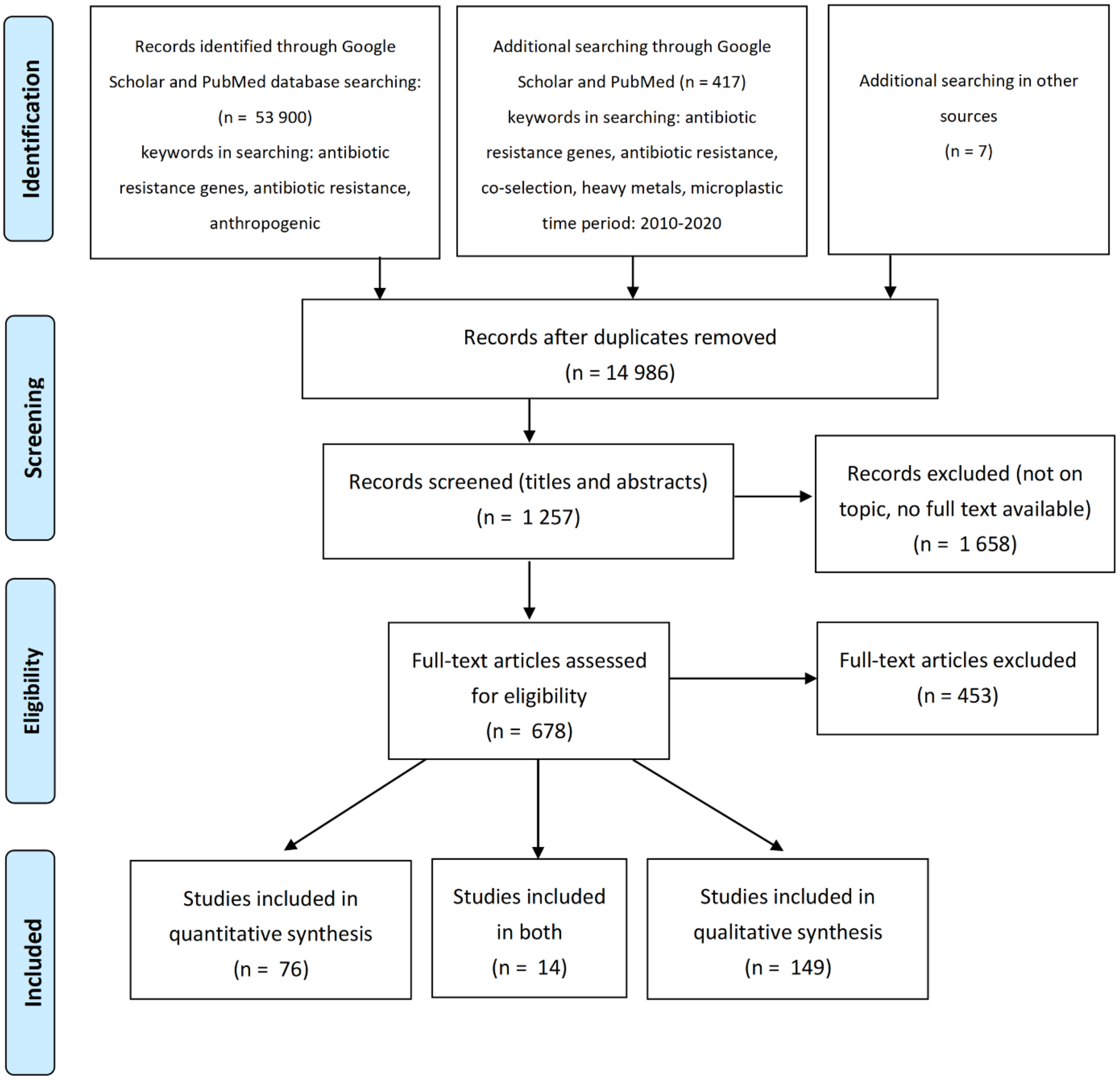

From: Moher D, Liberati A, Tetzlaff J, Altman DG, The PRISMA Group (2009). *P*referred *R*eporting *I*tems for *S*ystematic Reviews and *M*eta-*A*nalyses: The PRISMA Statement. PLoS Med 6(7): e1000097. doi:10.1371/journal.pmed1000097

For more information, visit www.prisma-statement.org.

Figure S1. PRISMA flowchart showing the results of the publication's search and screening process for this review.

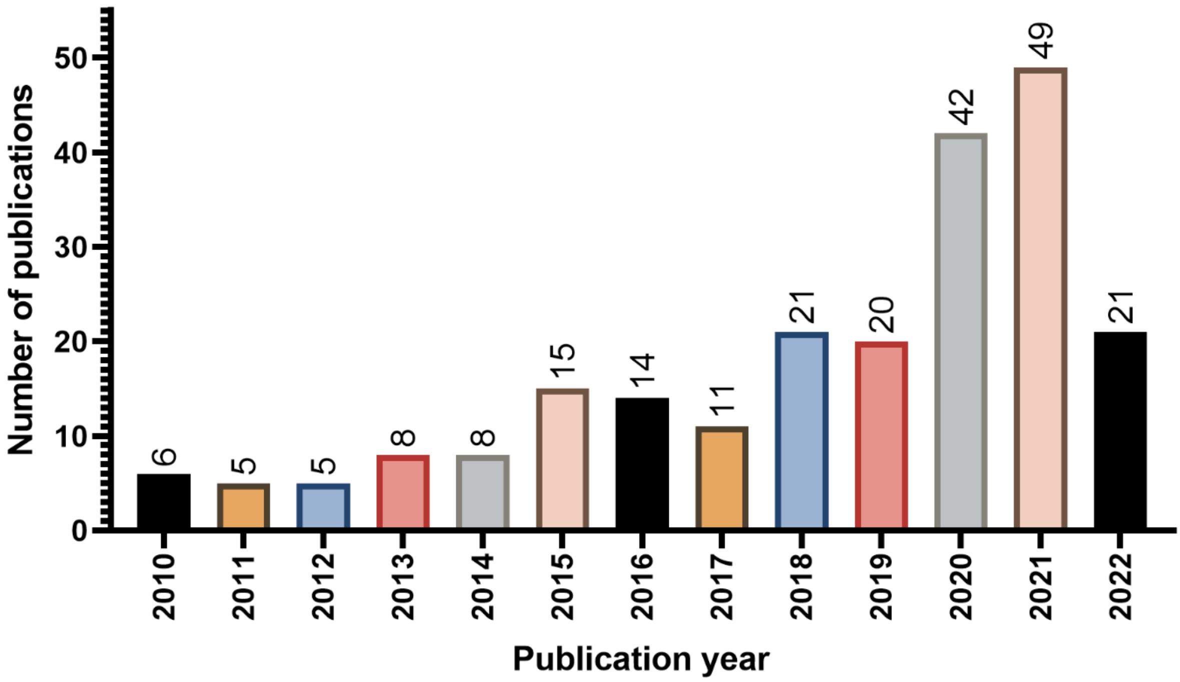

Figure S2. The number and date of publications used to develop this review.
